# High-mobility group box 1 is associated with neurological outcome in patients with post-cardiac arrest syndrome after out-of-hospital cardiac arrest

**DOI:** 10.1186/s40560-016-0161-4

**Published:** 2016-05-31

**Authors:** Taku Omura, Shigeki Kushimoto, Satoshi Yamanouchi, Daisuke Kudo, Noriko Miyagawa

**Affiliations:** Department of Emergency and Critical Care Medicine, Tohoku University Hospital, 1-1 Seiryo-machi, Aoba-ku, Sendai, 980-8574 Japan; Division of Emergency and Critical Care Medicine, Tohoku University Graduate School of Medicine, 1-1 Seiryo-machi, Aoba-ku, Sendai, 980-8574 Japan; Emergency Centre, Osaki Citizen Hospital, 3-8-1 Honami, Furukawa, Osaki, 989-6183 Japan

**Keywords:** Post-cardiac arrest syndrome, High-mobility group box 1, Mitochondrial DNA, Alarmin

## Abstract

**Background:**

Alarmins, including high-mobility group box 1 (HMGB-1), can be released from damaged tissues and activated cells as inflammatory mediators. We aimed to evaluate HMGB-1 and mitochondrial DNA dynamics and estimate the prognostic value for neurological outcome in patients with post-cardiac arrest syndrome after out-of-hospital cardiac arrest.

**Methods:**

We evaluated the dynamics of HMGB-1, mitochondrial DNA, and other variables in patients with return of spontaneous circulation after out-of-hospital cardiac arrest. Patients were divided into two groups according to the cerebral performance category at 30 days: the favourable outcome group (cerebral performance categories 1 and 2) and unfavourable group (≥3).

**Results:**

Twenty-one patients were included, and 11 demonstrated favourable outcomes. HMGB-1 levels and mitochondrial DNA on day 1 were significantly higher than on days 2, 3, 5, and 7. Plasma levels of HMGB-1 on day 1 correlated with prognostic parameters (estimated interval to return of spontaneous circulation, lactate, and NH3), tissue damage, systemic inflammation, and disease severity. HMGB-1 on day 1 in the unfavourable group was significantly higher than in the favourable group (median [interquartile range] 15.5 [6.65–18.7], 39.4 [17–69.5], *P* = 0.009). These findings were not observed regarding mitochondrial DNA. Regarding HMGB-1 prediction accuracy for a good neurological outcome, the area under the receiver operating characteristic curve was 0.864 (95 % confidence interval 0.702, 1.000).

**Conclusions:**

HMGB-1 may be involved in acute-phase post-cardiac arrest syndrome pathophysiology, and an increase in plasma levels may be associated with a poor neurological outcome. The study was registered with the University Hospital Medical Information Network Clinical Trials Registry ID: UMIN000006714.

**Electronic supplementary material:**

The online version of this article (doi:10.1186/s40560-016-0161-4) contains supplementary material, which is available to authorized users.

## Background

In 2008, post-cardiac arrest syndrome (PCAS) was proposed as a new term that incorporates all pathophysiological processes occurring after a return of spontaneous circulation after cardiac arrest [1]. There are four elements to PCAS: (1) hypoxic brain injury, (2) myocardial dysfunction, (3) systemic ischemia/reperfusion response, and (4) persistent precipitating pathology. In particular, systemic ischemia/reperfusion causes systemic inflammatory response syndrome (SIRS), which can lead to organ damage and multiple organ dysfunction in patients with PCAS [1]. Although these pathophysiological changes are caused by non-infectious inflammation, they demonstrate a clinical presentation similar to sepsis [2]. Molecules known as pathogen-associated molecular patterns (PAMPs) have been demonstrated to play a crucial role in the pathophysiology of sepsis [3]. In the pathophysiology of non-infectious inflammatory conditions such as trauma and burns, internal and endogenous molecules are released from damaged tissues and promote immunity, coagulation, and inflammatory responses [4]. These molecules are called “alarmins” [5]. In SIRS associated with PCAS, it has been suggested that alarmins may cause a non-infectious systemic inflammatory response [2, 6].

Recently, the pathophysiological roles of high-mobility group box 1 (HMGB-1) and mitochondrial DNA (mtDNA) were reported as alarmins in critical conditions [4, 7–11]. However, the roles of these molecules in PCAS have not been elucidated clearly. Therefore, the purpose of this study was to evaluate HMGB-1 and mtDNA dynamics, the relationship between HMGB-1, mtDNA, and severity of conditions and estimate the prognostic values of these molecules in patients with post-cardiac arrest syndrome after out-of-hospital cardiac arrest.

## Methods

### Patients

We performed a prospective, observational study of patients admitted to an academic tertiary care emergency centre from August 2011 to December 2012. The inclusion criteria were patients who were unresponsive after a return of spontaneous circulation (ROSC) after out-of-hospital cardiac arrest. Cardiac arrest was defined as the absence of spontaneous respiration, a palpable pulse, or responsiveness to stimuli. The exclusion criteria were as follows: trauma, age under 18 years, previous completion of a “Do Not Attempt Resuscitation (DNAR)” form, a Glasgow coma scale (GCS) score >8 at hospital arrival, and the presence of primary brain pathologies. The ethics committee of the institution approved this study (approval reference number: 2011-264), and all study subjects provided written informed consent. The study was registered with the University Hospital Medical Information Network Clinical Trials Registry (UMIN-CTR ID: UMIN000006714).

### Procedures after hospital arrival

Clinical management of OHCA and PCAS was performed according to the 2010 American Heart Association Guidelines for Cardiopulmonary Resuscitation and Emergency Cardiovascular Care [12]. If refractory shock from conventional intensive care was not sustained, we initiated therapeutic hypothermia.

### Data collection

All data were collected prospectively. Age, sex, type of bystander-witness status, bystander-initiated cardiopulmonary resuscitation (CPR), origin of cardiac arrest, initially documented electrocardiogram (ECG) rhythms, and the interval from collapse to ROSC were collected. All blood samples from patients were taken at the same time on arrival at the hospital and on the second, third, fifth, and seventh day after ROSC. Blood samples were centrifuged at 3000 rpm for 10 min, and the supernatants were stored in a −80 °C refrigerator until the analyses. HMGB-1 concentrations were measured using an enzyme-linked immunosorbent assay (Shino-Test Corporation, Sagamihara, Kanagawa, Japan). The lower sensitivity limit for HMGB-1 was 1 ng ml^−1^ and cross-reactivity with HMGB-2 was less than 10 %.

DNA isolation from plasma and mtDNA quantification by real-time quantitative polymerase chain reaction (PCR) were performed as previously described [11]. We centrifuged blood samples at ×1600 rpm for 10 min and then filtered the plasma through a 0.22-μm filter to remove platelets and cellular fragments bound to mtDNA. DNA isolation from filtered plasma samples and quantification of mtDNA by real-time quantitative PCR (qPCR) were performed [4]. Real-time PCR standard curves were created to quantify the mtDNA concentration by using purified mtDNA. The concentration of mtDNA was determined with a spectrophotometer. The purified mtDNA contained <0.1 % nuclear genomic DNA as determined using qPCR. The concentrations of mtDNA were calculated and expressed as micrograms in 1 ml of plasma. The results of biomarker levels were not blinded for outcome assessments.

The severity of illness was evaluated according to the Acute Physiology and Chronic Health Evaluation (APACHE)-II score on the day of enrolment [13]. A diagnosis of disseminated intravascular coagulation (DIC) was made on the basis of the scoring system of the Japanese Association for Acute Medicine (JAAM) [14] and the International Society on Thrombosis and Haemostasis (ISTH) [15].

### Data analysis and outcomes

The patients’ neurological outcomes were provided by Glasgow-Pittsburgh cerebral performance categories (CPCs) [16] at 30 days after OHCA. The patients were divided into two groups according to CPC at 30 days after OHCA: the favourable neurological outcome group (CPC = 1 or 2) and the unfavourable group (CPC ≥ 3).

Data are expressed as medians and interquartile ranges or as the number and percentage. All statistical analyses were performed using SPSS 22 for Windows (SPSS, Chicago, IL, USA). Comparisons between the two groups were performed using the Mann-Whitney *U* test, and categorical variables were compared between the groups using either Pearson’s chi-square or Fisher’s exact test, as appropriate. Correlations between HMGB-1, mtDNA, and other variables were performed using Spearman’s rank correlation test. The Kruskal-Wallis one-way analysis of variance was used for multiple comparisons between groups, and *P* values were adjusted with a Bonferroni correction for multiple testing. We evaluated the predictive precision of each parameter using the area under the receiver operating characteristic (ROC) curve (AUC). The cutoff values were determined based on the value that maximized the Youden index. Differences with a *P* value <0.05 were considered to be statistically significant. Furthermore, *P* < 0.005 (after Bonferroni correction) was used for testing multiple comparisons between groups.

## Results

### Characteristics of patients with ROSC after OHCA

**Fig. 1:**
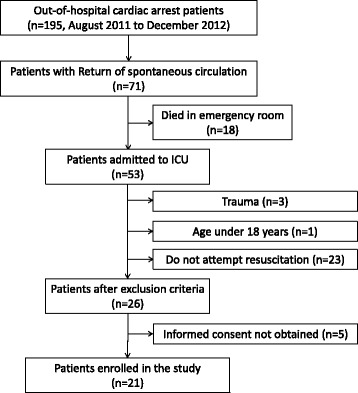
Flow chart of the study population. *ICU* intensive care unit

One hundred and ninety-six patients arrived on our hospital after out-of-cardiac arrest (Fig. 1). Seventy-one patients achieved return of spontaneous circulation, and 53 of 71 patients were admitted to intensive care unit. Twenty-one patients were enrolled in this study after the exclusion criteria were applied. All patient data are included in Additional file 1. The characteristics of the 21 enrolled patients and comparisons of the 11 patients with favourable outcomes with the 10 patients with unfavourable outcomes are demonstrated in Table 1. Ten of 11 patients in the favourable group and 7 of 10 in the unfavourable group were cardiac origin. Although 8 of 11 rhythms initially documented on the scene were shockable in the favourable group, only 3 of 10 were shockable in the unfavourable group; therapeutic hypothermia was applied in 9 (81.8 %) and 5 (50 %) cases, respectively. The estimated interval from the collapse to ROSC, initial levels of lactate and NH_3_, and interleukin-6 concentrations at the time of admission were different between the groups.

**Table 1:** Characteristics and physiology of patients after out-of-hospital cardiac arrest

Characteristic	All patients (*n* = 21)	Favourable group (*n* = 11)	Unfavourable group (*n* = 10)	*P* value
Age—years, median (IQR)	62.0 (47.0–79.0)	67.0 (36.0–76.0)	58.5 (47.0–83.0)	0.647
Male sex—no. (%)	13 (61.9 %)	8 (72.7 %)	5 (50 %)	0.387
Type of bystander-witness status—no. (%)	16 (76.2 %)	8 (72.7 %)	8 (80 %)	1.000
Bystander-initiated CPR—no. (%)	14 (66.7 %)	7 (72.7 %)	7 (70 %)	1.000
Cardiac origin—no. (%)	17 (81.0 %)	10	7	0.311
Initially documented rhythms on the scene of cardiac arrest—no.				0.123
VF/pulseless VT	11	8	3	
Non-VF/pulseless VT	10	3	7	
Estimated interval from collapse to ROSC—min	16.5 (13.0–29.5)	13.5 (10.0–16.25)	23.5 (16.75–44.25)	0.014
Therapeutic hypothermia—no. (%)	14 (66.7 %)	9 (81.8 %)	5 (50 %)	0.183
Initial lactate—mmol L^−1^		3.9 (2.4–7.5)	12.1 (7.08–15.70)	0.003
Initial NH_3_—μg dl^−1^		90.0 (41.0–140.0)	221.0 (90.5–345.5)	0.025
Myoglobin (day 1)—ng ml^−1^		402 (95–1343)	2008 (323–8358)	0.070
APACHE-II		24 (15–29)	30 (26–37)	0.072
IL-6 (day 1)—pg ml^−1^		23.5 (13.9–53.9)	88.0 (56.2–687.5)	0.024
Max IL-6—pg ml^−1^		84.9 (45.8–119.0)	196.5 (82.4–30637.5)	0.087
Max JAAM DIC		3.0 (2.0–4.0)	4.0 (2.0–6.25)	0.285
Max ISTH DIC		3.0 (2.0–3.0)	3.0 (2.75–5.5)	0.338
Max SOFA		10 (7.0–11.0)	10.0 (8.75–13.5)	0.499
Max d-dimer		7.0 (4.9–25.1)	24.7 (4.9–25.1)	0.074
Neurological outcomes 30 days after OHCA				
Survival (CPCs 1–4)—no. (%)	14 (66.7 %)			
CPC 1, good performance	9			
CPC 2, moderate disability	2			
CPC 3, severe disability	1			
CPC 4, vegetative state	2			
CPC 5, death	7			

*Abbreviations*: *CPR* cardiopulmonary resuscitation, *VF* ventricular fibrillation, *VT* ventricular tachycardia, *ROSC* return of spontaneous circulation, *OHCA* out-of-hospital cardiac arrest, *CPC* cerebral performance category scale, *APACHE* Acute Physiology and Chronic Health Evaluation, *IL* interleukin, *JAAM* Japanese Association for Acute Medicine, *ISTH* International Society of Thrombosis and Haemostasis, *DIC* disseminated intravascular coagulation, *SOFA* Sequential Organ Failure Assessment

### Plasma levels of HMGB-1 and mtDNA

**Fig. 2:**
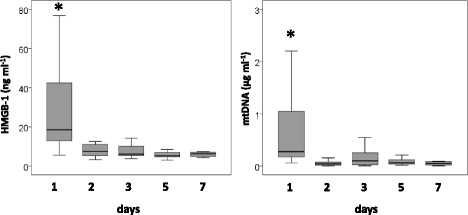
Changes in plasma levels of high-mobility group box 1 and mitochondrial DNA in patients after out-of-hospital cardiac arrest with return of spontaneous circulation. Both of these concentrations were significantly higher on day 1 than on days 2, 3, 5, and 7. Kruskal-Wallis one-way analysis of variance with a Bonferroni correction for multiple testing, *P < 0.001 vs. all other days. *HMGB-1* high-mobility group box 1, *mtDNA* mitochondrial DNA

**Fig. 3:**
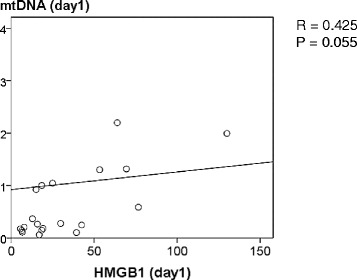
Relationship between high-mobility group box 1 plasma levels and mitochondrial DNA in patients after out-of-hospital cardiac arrest with return of spontaneous circulation. Spearman’s rank correlation coefficient was used. *R* = 0.425, *P* = 0.055. *HMGB-1* high-mobility group box 1, *mtDNA* mitochondrial DNA

Changes in the patients’ plasma levels of HMGB-1 and mtDNA are shown in Fig. 2. Both of these concentrations were significantly higher on day 1 than on days 2, 3, 5, and 7 (*P* < 0.001). However, there was no significant correlation between plasma HMGB-1 levels and mtDNA on day 1 (*R* = 0.425, *P* = 0.055) (Fig. 3).

### Relationship between HMGB-1, mtDNA, and clinical variables

Plasma levels of HMGB-1 on the day of admission correlated with parameters associated with resuscitation/prognosis (estimated interval from collapse to ROSC, lactate, and NH_3_ levels), tissue damage (myoglobin), systemic inflammation (interleukin-6), and severity of condition (APACHE-II and DIC scores) (Table 2). On the contrary, there was no correlation between plasma levels of mtDNA and these variables, except for the initial blood lactate concentration.

**Table 2:** Relationship between plasma levels of high-mobility group box 1, mitochondrial DNA, and physiologic parameters in patients after out-of-hospital cardiac arrest with return of spontaneous circulation

	HMGB-1 (day 1)		mtDNA (day 1)	
	*r*	*P* value	*r*	*P* value
Estimated interval from collapse to ROSC	0.528	0.017	0.108	0.651
Initial lactate	0.715	0.000	0.463	0.034
Initial NH_3_	0.674	0.001	0.168	0.478
Myoglobin (day 1)	0.555	0.011	0.310	0.184
APACHE-II	0.468	0.032	−0.315	0.559
IL-6 (day 1)	0.457	0.037	0.108	0.642
Maximal JAAM DIC	0.595	0.004	0.059	0.799
Maximal ISTH DIC	0.504	0.020	0.087	0.708

*Abbreviations*: *ROSC* return of spontaneous circulation, *APACHE-II* Acute Physiology and Chronic Health Evaluation II, *IL* interleukin, *JAAM* Japanese Association for Acute Medicine, *ISTH* International Society of Thrombosis and Haemostasis, *DIC* disseminated intravascular coagulation, *SOFA* Sequential Organ Failure Assessment, *mtDNA* mitochondrial DNA, *HMGB-1* high-mobility group box 1

### Plasma levels of HMGB-1, mtDNA, and neurological outcomes

**Fig. 4:**
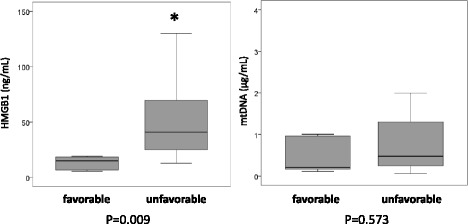
Plasma levels of high-mobility group box 1 and mitochondrial DNA in patients with favourable and unfavourable neurological outcomes after out-of-hospital cardiac arrest. *HMGB-1* high-mobility group box 1, *mtDNA* mitochondrial DNA

Plasma levels of HMGB-1 in patients with the unfavourable neurological outcome group were significantly higher on day 1 than in the favourable outcome group (*P* = 0.009). However, no significant difference between plasma mtDNA levels in the favourable and unfavourable groups on day 1 (*P* = 0.573) (Fig. 4).

**Fig. 5:**
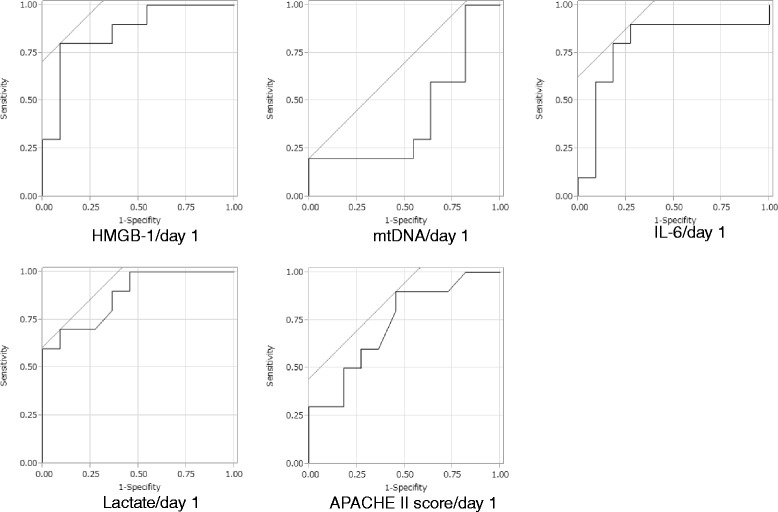
Prognostic value of high-mobility group box 1, mitochondrial DNA, interleukin-6, and physiologic variables in patients with favourable and unfavourable neurological outcomes after out-of-hospital cardiac arrest. The areas under the receiver operating characteristic curves for high-mobility group box 1, mitochondrial DNA, interleukin-6, initial lactate, and Acute Physiology and Chronic Health Evaluation II score were 0.864, 0.573, 0.791, 0.877, and 0.732, respectively. The optimal cutoff value for high-mobility group box 1 was 22 ng ml^−1^ with a specificity of 0.90 and a sensitivity of 0.80. *HMGB-1* high-mobility group box 1, *mtDNA* mitochondrial DNA, *APACHE-II* Acute Physiology and Chronic Health Evaluation II

We evaluated the accuracy of the prognostic value of HMGB-1, mtDNA, interleukin-6, initial lactate, and APACHE-II score using ROC curve analyses (Fig. 5); the AUCs were 0.864, 0.573, 0.791, 0.877, and 0.732, respectively. The optimal cutoff value for HMGB-1 was 22 ng ml^−1^ and demonstrated a specificity of 0.90 and a sensitivity of 0.80.

## Discussion

Our study elucidates the HMGB-1 and mtDNA dynamics in patients with PCAS after OHCA and suggests that plasma levels of HMGB-1 on the day of admission after ROSC might be the prognostic values for neurological outcomes in patients with PCAS after OHCA.

Although mtDNA has been recognized as one of the alarmins and showed similar serial changes to those of HMGB-1 in patients with PCAS after OHCA, plasma levels of mtDNA on the day of admission did not correlate with parameters associated with prognosis, tissue damage, or disease severity, and the values were not different between the favourable and unfavourable outcome groups. In previous study, we demonstrated that changes in plasma HMGB-1 and mtDNA levels in patients with severe trauma and sepsis [11]. Although plasma levels of HMGB-1 and mtDNA on the day of admission were higher than those of the other days, which decrease gradually during the initial 7 days, in patients with severe trauma, plasma HMGB-1 and mtDNA were sustained in elevated levels in patients with sepsis. Present study showed that the dynamics of plasma HMGB-1 and mtDNA levels were similar to those in patients with trauma. However, relationship between the plasma levels of HMGB-1, mtDNA, and disease severities and outcomes in patients with PCAS were different from those of severe trauma. Therefore, it is suggested that the role of HMGB-1 and mtDNA in the pathogenesis of PCAS after OHCA, including brain injuries, might be different from those in severe trauma.

HMGB-1 is known as a late mediator in sepsis [7]. Moreover, it has been reported that HMGB-1 levels increased in the early phase of trauma and correlated with the severity of injury [9, 17]. Active leakage (secretion) and passive leakage are known as mechanisms that increase plasma levels of HMGB-1 [7, 8, 18], and these mechanisms may contribute differently to the pathophysiologies of sepsis and trauma. Active secretion occurs when HMGB-1 is actively released extracellularly from the stimulation of tumour necrosis factor-α, interferon-γ, and other inflammatory mediators in sepsis [19–25]. Passive leakage is caused by the necrosis of injured cells due to trauma, infections, and tissue ischemia [8, 26–28]. On the contrary, the mechanism of mtDNA release has been suggested to be passive discharge into the extracellular space from injured mitochondria in the necrotic cell [10]. Therefore, the pathophysiological role and clinical significance may differ between HMGB-1 and mtDNA in various critical conditions.

PCAS is a complex pathologic condition and includes ischemic tissue damage and SIRS, leading to multiple organ dysfunction [2, 6]. Although HMGB-1 can be released as a consequence of ischemic injury, it may also play a role as an early trigger of inflammation and may be involved as a late modulator of inflammation. HMGB-1 induces the release of proinflammatory cytokines, and then promotes further inflammation in the brain [29, 30]. Therefore, an increase in plasma levels of HMGB-1 may be involved in the pathogenesis of a poor neurological outcome and post-resuscitation inflammatory status. In recent study, an association between HMGB-1 in cerebrospinal fluid and neurological outcomes in patients with PCAS after OHCA was reported [31]. In contrast, serum HMGB-1 levels did not correlated to neurological outcomes. However, the study may consist of small sample size with only seven patients of the favourable neurological outcome, which may be insufficient to evaluate the prognostic value adequately. As demonstrated in this study, plasma levels of HMGB-1 on the day of admission might have the prognostic value in patients with PCAS after OHCA. To clarify the pathophysiological role of HMGB-1 in patients with PCAS, further large-scale study is required.

This study has some limitations. The main limitations are the observational study design, small sample size, and the distribution of aetiologies in cardiac arrest and neurological severity levels. In addition, although there are some confounding factors with respect to the levels of HMGB-1 and mtDNA, and clinical impacts of these mediators exist, their influences have not been adjusted because of the limited number of patients. Further evaluations in a larger number of patients are therefore needed to determine whether HMGB-1 can be a reliable biomarker predicting neurological outcome and to clarify its pathophysiological significance.

## Conclusions

HMGB-1 may be involved in the pathophysiology of systemic inflammation and neurological sequences in patients with PCAS after OHCA and an increase in plasma levels may be associated with a poor neurological outcome. To verify the role of HMGB-1, further evaluations in a larger number of patients are necessary.

## Availability of supporting data

The datasets supporting the conclusions of this article are included within the article and its additional file.

## Abbreviations

APACHE-II, Acute Physiology and Chronic Health Evaluation II; DIC, disseminated intravascular coagulation; DNAR, Do Not Attempt Resuscitation; GCS, Glasgow coma scale; HMGB-1, high-mobility growth box 1; mtDNA, mitochondrial DNA; OHCA, out-of-hospital cardiac arrest; PAMP, pathogen-associated molecular pattern; PCAS, post-cardiac arrest syndrome; ROSC, return of spontaneous circulation; SIRS, systemic inflammatory response syndrome

## Additional file

Additional file 1: Patient data.
